# The use of a darunavir/ritonavir once-daily regimen in two pregnant women

**DOI:** 10.1186/1758-2652-13-S4-P189

**Published:** 2010-11-08

**Authors:** JS Lambert, LJ Else, V Jackson, L Dickinson, DJ Back, M Brennan, C Weldridge, S Coulter-Smith, S Gibbons, SH Khoo

**Affiliations:** 1 The Rotunda Hospital, Parnell Square, Dublin, Ireland; 2 University of Liverpool, Department of Pharmacology, Liverpool, UK; 3 The Mater Misericordiae University Hospital, Infectious Diseases, Dublin, Ireland

## Purpose of the study

Darunavir (DRV) is a second generation protease inhibitor (PI), first licensed for use in 2006. It is indicated for use in a treatment experienced population and is effective in vitro against both wild type and PI-resistant HIV. As a relatively new drug there is little published information on the pharmacokinetics of darunavir in pregnancy. Here we examine the pharmacokinetics of darunavir/ritonavir [DRV/r 800/100mg once daily (OD)] in two women, over the course of pregnancy and postpartum.

## Methods

A prospective open labelled study was established to enrol HIV positive pregnant women on DRV/r as part of their routine maternity care. DRV plasma trough concentrations were determined in the first (T1) and/or second (T2) and/or third (T3) trimester using a validated HPLC-MS/MS methodology with a limit quantification of 16 ng/ml. Postpartum (PP) sampling was also performed.

## Summary of results

To date two women have been recruited. Both were black African and initiated treatment prior to pregnancy. Each woman was virally suppressed (HIV RNA <50 cpm) throughout pregnancy and had CD4 cell counts >300 cells/mm^3^ (range 341-470). Patient 1 had a TDM sample drawn in each trimester and at PP. DRV concentrations in T1 [3790ng/ml], T2 [1285ng/ml] and T3 [1773ng/ml] were considerably (27-75%) lower relative to concentrations at PP [5227ng/ml]. Patient 2 had TDM samples taken in T2 and PP only. Again a considerably lower (~72%) DRV concentration was noted at T2 compared with PP. In both cases, DRV concentrations in pregnancy and postpartum were above the accepted minimum effective concentration for wt virus (MEC; 550 ng/ml, based on in vitro studies).

## Conclusions

In the two cases examined, DRV/r (800/100mg, OD) was effective at achieving adequate therapeutic drug levels (>550ng/ml) during pregnancy. However, reduced DRV plasma concentrations in the second/third trimesters, highlights the need for TDM in this population, and warrants further study of pregnancy-associated changes in DRV pharmacokinetics.

